# Novel Use of Manganese Gluconate as a Marker for Visualization of Tablet Dissolution in the Fed Human Stomach Using Magnetic Resonance Imaging

**DOI:** 10.1021/acs.molpharmaceut.4c00854

**Published:** 2024-12-05

**Authors:** Tejal Akbar, Pavel Gershkovich, Konstantinos Stamatopoulos, Penny A. Gowland, Snow Stolnik, James Butler, Luca Marciani

**Affiliations:** 1 Nottingham Digestive Diseases Centre and National Institute for Health Research (NIHR), Nottingham Biomedical Research Centre, Nottingham University Hospitals NHS Trust and University of Nottingham, Nottingham NG7 2UH, U.K.; 2 School of Pharmacy, University of Nottingham, Nottingham NG7 2RD, U.K.; 3 Drug Product Development, GSK R&D, Ware, Hertfordshire SG12 0GX, U.K.; 4 Sir Peter Mansfield Imaging Centre, School of Physics and Astronomy, University of Nottingham, Nottingham NG7 2QX, U.K.

**Keywords:** *in vivo*, oral drug delivery, magnetic resonance imaging (MRI), dissolution, food interactions, food effects

## Abstract

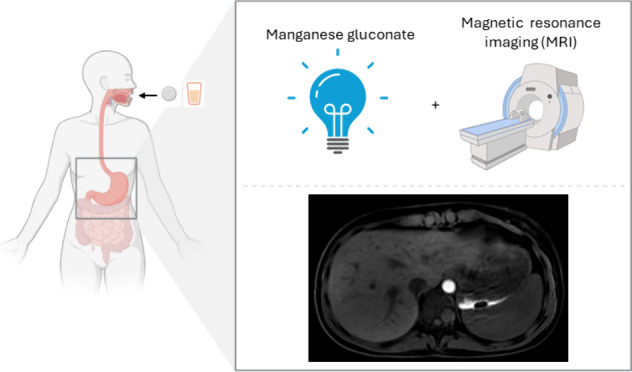

Magnetic resonance imaging (MRI) of dry or solid materials in the gastrointestinal (GI) tract requires the use of contrast agents to enhance visualization of the dosage forms. In this study, we explore the novel use of manganese gluconate added to tablets. Manganese was released during tablet dissolution, generating a bright “halo” effect around the tablets, consistent with shortening of the longitudinal relaxation time of the bulk water surrounding the tablet. This is the first study to use MRI to directly image tablet dissolution in the fed stomach using a manganese gluconate contrast agent as dissolution marker.

## 1 Introduction

The addition of a contrast agent into a drug dosage form significantly enhances its visibility when imaging it in the GI tract using MRI.^1^ The contrast agent assists to discriminate the system from water, food materials, and gas present in the complex GI environment. In recent years, contrast agents including gadolinium-chelates^2^ and magnetite and manganese^3−7^ have been attempted not only in imaging the drug dosage form but also in imaging its disintegration and dispersion in both the fasted and fed stomach. The ease at which these contrast agents, especially magnetite, are inserted into a capsule shell has encouraged focus on the development in testing of capsule-based technological advancements.^8−10^ However, capsule shell disintegration time has been problematic to determine using magnetite, due to the large nature of its susceptibility artifact.^5,11,12^ Previous studies have investigated the use of contrast agents embedded in a tablet as the drug dosage form. Most notably, Steingoetter et al.^2^ used paramagnetic gadolinium (Gd) chelates embedded into a slow release floating tablet to serve as a water-soluble drug model. Gd distribution profiles within the fed stomach were determined using MRI. However, the use of Gd compounds for research purposes has recently been discouraged over safety concerns.^13^ In this study, manganese gluconate is explored as an alternative contrast agent to overcome problems associated with previously used contrast agents.

Manganese is a non-lanthanide metal which is crucial in cell biology.^15^ It is often present in food and is generally regarded as safe (GRAS). Manganese shortens the T1 relaxation time when dissolved in water when imaging using MRI. This leads to a “positive” contrast enhancement or a “bright” appearance on a T1 weighted MRI image. For instance, manganese in various forms has featured in previous MRI studies imaging dosage forms in the GI tract. Manganese ions present in dried and sugared pineapple were successfully added to floating and sinking acid-resistant capsules to image their transit and disintegration behavior.^5^ The varying water consistency between batches and producers of dried pineapple showed differing signal intensities for different samples leading to repeatability and reliability issues in further studies. Additionally, hibiscus tea powder,^6^ which contains manganese, was encapsuled in different hard-shell capsule combinations, but there were difficulties in replicating *in vitro* results in human studies. A similar problem was observed in another study when manganese gluconate dihydrate was combined with black iron oxide inside a targeted release capsule formulation. Manganese dispersion could not be observed,^16^ possibly due to low water availability in the distal bowel and competing artifacts from the iron presence.

While manganese gluconate has been used previously as a positive contrast agent for MRI imaging in the GI tract,^14^ to our knowledge this is the first study to directly image the dissolution of manganese from a tablet in the fed stomach using MRI. It is well-known that one of the rate limiting steps to absorption of drugs from the GI tract to the systemic circulation is dissolution.^15^ This area has not been actively investigated due to limitations posed by contrast agents.

## 2 Materials and Methods

### 2.1 Tablet Development

Immediate release tablets were manufactured using a 10-station research and development Riva Piccola rotary press equipped with 2 round, bevelled edge flat faced 15 mm punch and die sets. Each 750 mg tablet included 18.75 mg of manganese gluconate (Sigma-Aldrich) and commonly used excipients such as spray dried lactose (65%w/w) (Medisca UK), microcrystalline cellulose (10.5%w/w) (Fisher Scientific UK), and magnesium stearate (2%w/w) (Sigma-Aldrich).

Compression forces of up to 18 kN were used to produce tablets with a maximum hardness of 33 N. Standard pharmacopeial tests such as uniformity of mass and disintegration time were used to assess and evaluate the success of the tablet formulation and manufacturing process. The disintegration time of the tablets was determined at 37 °C in water using a USP apparatus (Erweka light, Germany). All tablets disintegrated in less than 12 min.

### 2.2 Experimental Design

The study was performed as an open label, single center study in healthy human adult subjects. Six participants were recruited and imaged (mean age 21 ± 0.8 years and body mass index 24.7 ± 4.8 kg/m^2^). Participants had no history of lactose intolerance, anemia, or abnormal liver function. The study protocol was approved by the University of Nottingham Research Ethics Committee, Approval Number 76-1123. All participants gave written informed consent before the study and had no contraindications to MRI. Each participant was asked to attend the study site in the morning after an overnight fast of at least 10 h. An initial baseline MRI scan was acquired to ensure that the stomach was indeed in a fasted state. After this, participants were asked to drink 300 mL of a nutrient drink to induce a fed state. This was prepared by mixing 85 g of Scandishake Mix (Nutricia) powder with 240 mL of water (428 kcal, 20.6 g of fat, 63.2 g of carbohydrates, 9 g of protein). Water was used instead of the recommended milk to reduce delays to tablet dissolution as a greater amount of water is then available to the tablet. After a period of 5 min, one tablet and 240 mL of water were administered to the participant while sitting in an upright position on the table of the MRI scanner. Time *t* = 0 min was defined as the time of tablet ingestion. MRI scans were then acquired serially for 1 h postadministration. The participants were asked to attend a second similar study approximately a week later. Four participants out of six returned, and the scanning process as described above was repeated. The remaining two participants did not return due to personal time commitments.

### 2.3 MRI Acquisition

A 3T GE Signa Premier MRI scanner (GE Healthcare) was used. Sagittal, coronal, and axial images were acquired using an abdominal receiver. Out of the total 10 MRI study days, 2 study days were conducted with the participant supine and 8 study days with the participants lying with the left side raised by approximately 30° using wedges to investigate the potential effects of gravity. A range of MRI sequences were used to image the stomach using short breath-holds to minimize respiratory motion. These included a T2-weighted fast imaging employing steady-state acquisition (FIESTA, slice thickness 7 mm, echo time 1.088 ms, flip angle 45°, repetition time 2.823 ms), a T1-weighted 3D liver acceleration volume acquisition flex sequence (3D LAVA Flex, slice thickness 4.4 mm, echo time 1.674 ms, flip angle 12°, repetition time 3.8 ms), and a dual echo sequence (slice thickness 6 mm, echo time 1.088 ms, flip angle 60°, repetition time 141.05 ms).

## 3 Results

**Figure 1:**
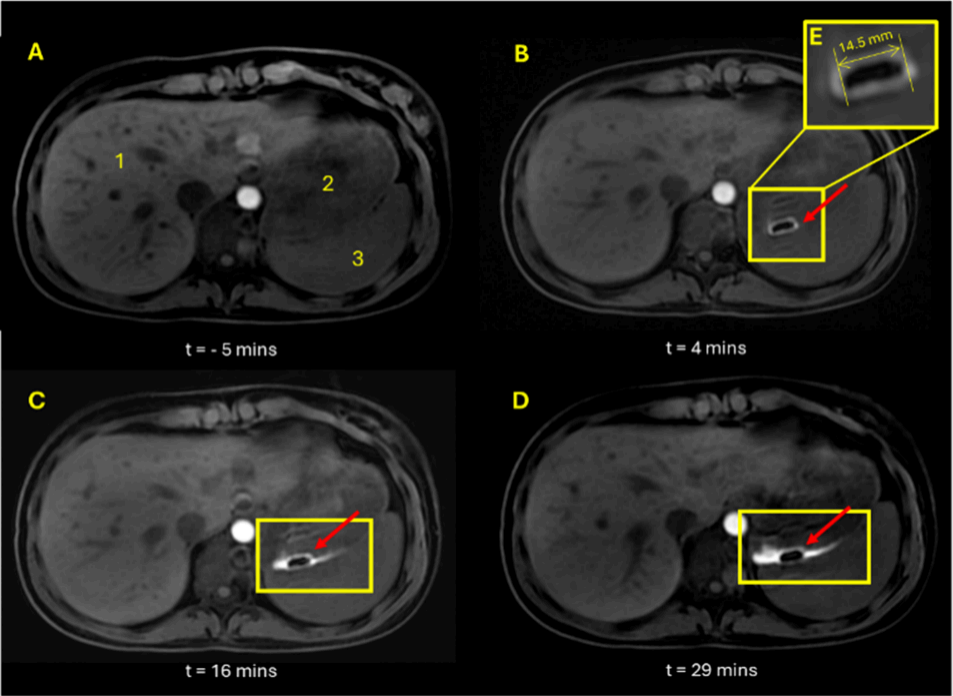
Axial, 3D LAVA MRI images of abdomen of participant 2. Image A shows image after administration of Scandishake drink and prior to tablet administration. Images B, C, and D show tablet at time points 4, 16, and 29 min, respectively, post tablet administration with inset E showing an enlarged image of tablet. Anatomical landmarks are indicated in image A: liver (1), stomach (2), spleen (3).

**Figure 2:**
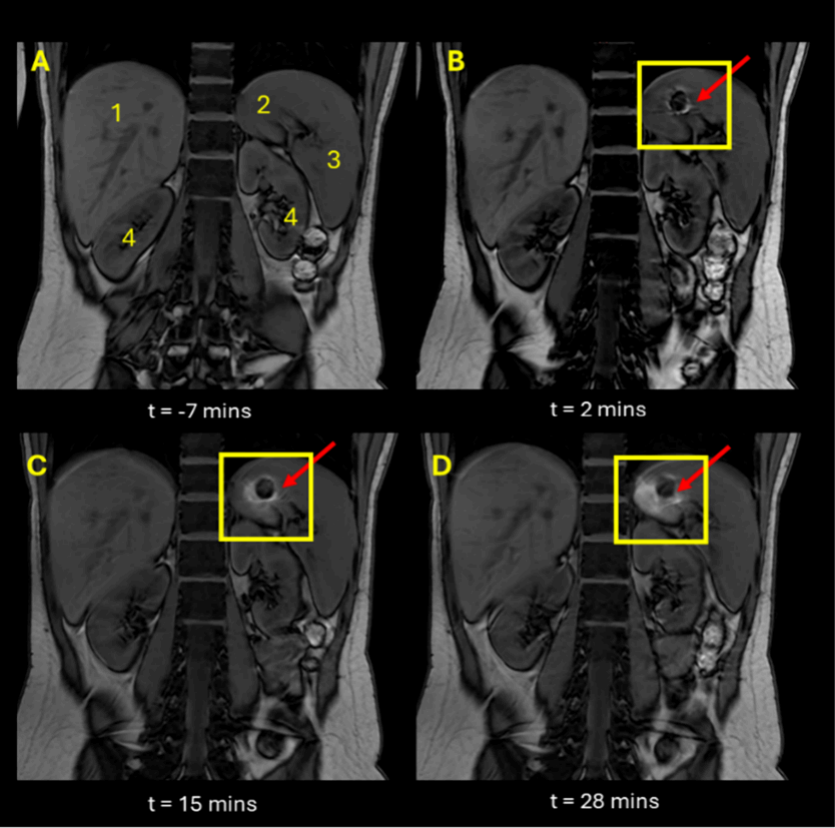
Coronal, T1-weighted dual echo MRI images of abdomen of participant 2 positioned supine with elevation of the left side. Image A shows image after administration of Scandishake drink and prior to tablet administration. Images B, C, and D show participant at time points 2, 15, and 28 min, respectively, post tablet administration. Anatomical landmarks are indicated in image A: liver (1), stomach (2), spleen (3), kidneys (4).

**Figure 3:**
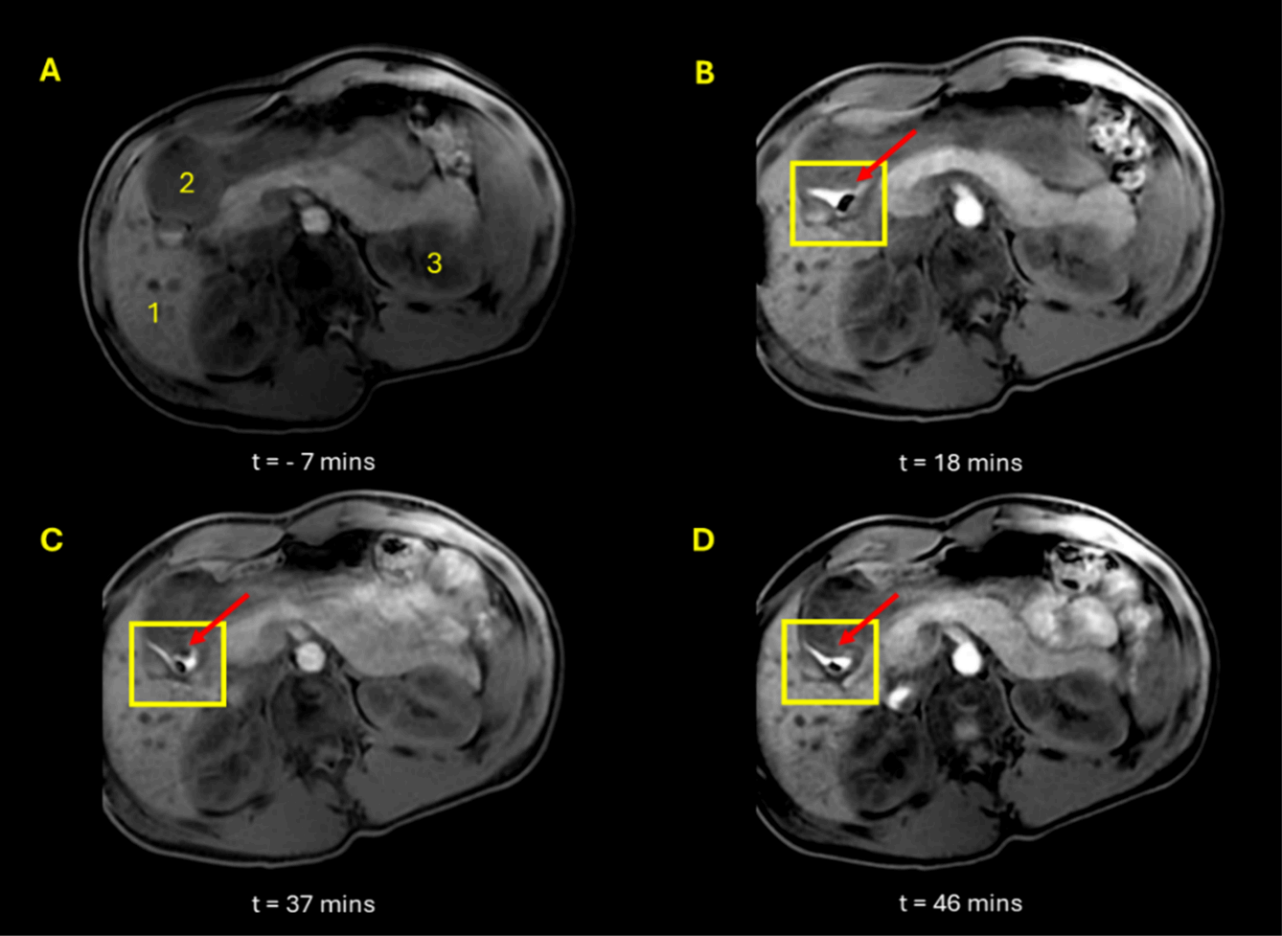
Axial, 3D LAVA MRI images of abdomen of participant 4 positioned supine with elevation of the left side. Image A shows image after administration of Scandishake drink and prior to tablet administration. Images B, C, and D show tablet at time points 18, 37, and 46 min, respectively, post tablet administration with inset E showing an enlarged image of tablet. Anatomical landmarks are indicated in image A: liver (1), stomach (2), spleen (3).

All participants tolerated the study procedures well and were able to swallow the tablet. Food and Drug Administration (FDA) guidance concerning tablet size and shape^16^ was followed to ensure ease of swallowing and minimal risk of adverse effects for participants. This study aimed at developing methods and showing proof-of-principle, and as such a relatively large sized tablet of 15 mm diameter was used. The ingested tablets were visible in the fed stomach in all 10 studies performed. The location of the tablets inside the stomach of the participants varied. After administration, the tablets were located in the fundus in 5 of the studies and in the antrum in the other 5 studies. When participants attended a second study day, the tablet did not always reside in the same position as the first study day. Figures 1B and 2B show the typical appearance of the tablet in the stomach of one participant 4 min after dosing. The participant was positioned in a supine position with no elevation. The tablet size and shape observed in the images are representative of the size and shape of the administered tablet; a bevelled edge tablet of approximately 15 mm in diameter is observed as shown in inset Figure 1E. The participants positioned with left side elevated at an angle encouraged the tablets to position more distally toward the antrum as demonstrated in Figure 3. The tilted body position in the scanner was not corrected on the images for evaluation. As such, the images are shown in the coronal plane with reference to the MRI scanner axis.

A bright “halo” effect can be observed around the surface of the tablet in Figures 1, 2, and 3. This is consistent with T1 relaxation time shortening of the bulk water surrounding the tablet due to dissolution of the manganese contrast agent in the stomach content. The “halo” remained largely around the tablet over the experimental time, suggesting that no significant mixing of the stomach contents occurred during the imaging period. With time, the tablets showed increased erosion, which was inferred by observing a decrease in tablet size and change in shape. The increase in signal from the meal contents around the tablet (seen as a larger bright area in the images) was also clearly seen to increase in size as the study progressed, as demonstrated in Figure 2. This phenomenon was observed consistently in all subjects in the T1-weighted imaging sequences. In 9 of the 10 studies, the tablets were visible in the images throughout the period of 1 h and appeared intact. In 1 study, the tablet however appeared to have emptied from the stomach after about 20 min postadministration. The other tablets remained in the stomach during the imaging period. The tablet was not detected anywhere else in the GI tract after this point.

## 4 Discussion

MRI has become an increasingly popular and powerful imaging tool for exploring drug dosage form interactions with food in the GI tract. In this study, it has been used to explore tablet dissolution. It is important to note that *in vivo* dissolution of a drug from oral formulations can also be evaluated by other methods such as pharmacokinetic markers, luminal fluid aspiration^17^ or alternative imaging techniques such as γ scintigraphy.^8^

The use of a “positive” contrast agent is demonstrated by Steingoetter et al.^2^ where Gd chelates were added to slow release tablets of similar dimensions to image the drug intragastric distribution in the fed stomach. That study was also able to visualize the tablet position and drug dispersion in the stomach with respect to the meal. Direct comparison of the dispersion characteristics of each contrast agent between the two studies, however, is not possible as they used differing tablet (slow release versus immediate release tablet) and meal compositions, influencing the dispersion characteristics of the incorporated contrast agents. The use of Gd is however currently discouraged for research studies due to recent safety concerns,^13,18^ therefore making manganese gluconate a more attractive option.

This is, to the best of our knowledge, the first study to use MRI to image tablet dissolution in the fed stomach using manganese gluconate as a contrast agent. As only one component of the tablet is being imaged, the appearance of the manganese contrast in the images may not adequately describe the entire tablet dissolution or be representative of the dissolution of the active pharmaceutical ingredient (API), particularly for poorly wetting and poorly soluble APIs. In this study, a bright “halo” effect is observed around the surface of the tablet, consistent with T1 shortening of the bulk water surrounding the tablet. This positive contrast enhancement or shortening of the T1 signal is observed only when the manganese from the tablet dissolves into water. The meal used here had low viscosity which, in conjunction with the inferred low mixing, may have been a contributing factor in making the “halo” visible over a prolonged time period. Stomach contractions may also have been present throughout the imaging period.

The MRI method could also be advantageous to quantify intrasubject variability in dissolution studies, which is a topic of great interest; methods to measure this would be advantageous in further studies. The use of manganese for inclusion in tablets for further exploratory studies has several implications including manganese gluconate’s stability to heat, humidity, and long-term storage. As manganese gluconate is slightly hygroscopic, it should be kept in a cool, dry place, away from direct sunlight in an airtight container. This should ensure it is stable over several years and not does start to degrade. Manganese gluconate, like other metal gluconates, can be subject to chelation interactions which can impact its bioavailability, absorption, and efficacy. For example, chelation of Mn ions with food components, such as phytates, can lead to a formation of insoluble complexes that can reduce and hinder absorption. The impact of this should be considered, especially when extending its use for investigating food effects using FDA recommended meals or other relevant meals.

Typically, for an oral formulation, dissolution of the drug must occur as the first step in ensuring an efficacious dose is delivered. Expanding knowledge of dispersion and dissolution using manganese gluconate has the potential for improved understanding of disintegration and dissolution from oral tablets, especially in the fed state, and may inform *in silico* modeling and potentially lead to better *in vitro* and *in vivo* agreement.

## 5 Conclusion

The dissolution of manganese gluconate added as an excipient in a tablet has been imaged using MRI. A brightening and “positive” contrast observed indicates tablet dissolution of manganese gluconate. This is a first step in investigating *in vivo* imaging of tablet performance using this approach and has the potential to be explored further in research studies involving various pre- and postprandial conditions.

## References

[ref1] Giovagnoni A.; Fabbri A.; Maccioni F. Oral contrast agents in MRI of the gastrointestinal tract. Abdominal Radiology 2002, 27 (4), 367 10.1007/s00261-001-0117-5. 12066234

[ref2] Steingoetter A.; et al. Magnetic resonance imaging for the in vivo evaluation of gastric-retentive tablets. Pharm. Res. 2003, 20, 2001–2007. 10.1023/B:PHAM.0000008049.40370.5a. 14725366

[ref3] Faas H.; et al. Monitoring the intragastric distribution of a colloidal drug carrier model by magnetic resonance imaging. Pharm. Res. 2001, 18, 460–466. 10.1023/A:1011098125916. 11451032

[ref4] Steingoetter A.; et al. Analysis of the meal-dependent intragastric performance of a gastric-retentive tablet assessed by magnetic resonance imaging. Alimentary Pharmacology & Therapeutics 2003, 18 (7), 713–720. 10.1046/j.1365-2036.2003.01655.x. 14510745

[ref5] Grimm M.; et al. Characterization of the gastrointestinal transit and disintegration behavior of floating and sinking acid-resistant capsules using a novel MRI labeling technique. European Journal of Pharmaceutical Sciences 2019, 129, 163–172. 10.1016/j.ejps.2019.01.012. 30639530

[ref6] Rump A.; et al. The effect of capsule-in-capsule combinations on in vivo disintegration in human volunteers: a combined imaging and salivary tracer study. Pharmaceutics 2021, 13 (12), 2002 10.3390/pharmaceutics13122002. 34959284 PMC8707024

[ref7] Seoane-Viano I.; et al. Visualizing disintegration of 3D printed tablets in humans using MRI and comparison with in vitro data. J. Controlled Release 2024, 365, 348–357. 10.1016/j.jconrel.2023.11.022. 37972762

[ref8] Akbar T.; et al. Use of Magnetic Resonance Imaging for Visualization of Oral Dosage Forms in the Human Stomach: A Scoping Review. Mol. Pharmaceutics 2024, 21 (4), 1553–1562. 10.1021/acs.molpharmaceut.3c01123. PMC10988553 38440796

[ref9] Kagan L.; et al. Gastroretentive accordion pill: enhancement of riboflavin bioavailability in humans. J. Controlled Release 2006, 113 (3), 208–215. 10.1016/j.jconrel.2006.03.022. 16806558

[ref10] Sager M.; et al. In vivo characterization of enTRinsic drug delivery technology capsule after intake in fed state: A cross-validation approach using salivary tracer technique in comparison to MRI. J. Controlled Release 2019, 313, 24–32. 10.1016/j.jconrel.2019.10.023. 31626859

[ref11] Rump A.; et al. In Vitro and In Vivo Evaluation of Carbopol 71G NF-Based Mucoadhesive Minitablets as a Gastroretentive Dosage Form. Mol. Pharmaceutics 2023, 20 (3), 1624–1630. 10.1021/acs.molpharmaceut.2c00835. 36705398

[ref12] Rump A.; et al. In Vivo Evaluation of a Gastro-Resistant HPMC-Based “Next Generation Enteric” Capsule. Pharmaceutics 2022, 14 (10), 1999 10.3390/pharmaceutics14101999. 36297435 PMC9609816

[ref13] Ramalho J.; et al. Gadolinium toxicity and treatment. Magn. Reson. Imaging 2016, 34 (10), 1394–1398. 10.1016/j.mri.2016.09.005. 27693607

[ref14] Grimm M.; et al. Enteric-Coated Capsules Providing Reliable Site-Specific Drug Delivery to the Distal Ileum. Mol. Pharmaceutics 2024, 21, 2828 10.1021/acs.molpharmaceut.3c01241. PMC11151196 38723178

[ref15] Hörter D.; Dressman J. Influence of physicochemical properties on dissolution of drugs in the gastrointestinal tract. Adv. Drug Delivery Rev. 2001, 46 (1–3), 75–87. 10.1016/S0169-409X(00)00130-7. 11259834

[ref16] Size, Shape, and Other Physical Attributes of Generic Tablets and Capsules. U.S. Department of Health and Human Services Food and Drug Administration Center for Drug Evaluation and Research (CDER), 2015.

[ref17] Augustijns P.; et al. Unraveling the behavior of oral drug products inside the human gastrointestinal tract using the aspiration technique: history, methodology and applications. European Journal of Pharmaceutical Sciences 2020, 155, 105517 10.1016/j.ejps.2020.105517. 32818656

[ref18] Fraum T. J.; et al. Gadolinium-based contrast agents: a comprehensive risk assessment. Journal of Magnetic Resonance Imaging 2017, 46 (2), 338–353. 10.1002/jmri.25625. 28083913

